# Association between burnout and physiological-psychological-social health domains among Chinese resident physicians: a network analysis approach

**DOI:** 10.1186/s12913-025-13200-y

**Published:** 2025-08-30

**Authors:** Jiarui Li, Tao Li, Chunfeng Xiao, Yanping Duan, Yinan Jiang, Wenqi Geng, Lili Shi, Xia Hong, Jianhua Du, Yinghan Xie, Jiaojiao Hu, Yufei Wang, Jinya Cao, Jing Wei

**Affiliations:** 1 Department of Psychological Medicine, Peking Union Medical College Hospital, Chinese Academy of Medical Sciences & Peking Union Medical College, 1 Shuai Fu Yuan, Dongcheng District, Beijing, 100730 China; 2 4+4 Medical Doctor Program, Chinese Academy of Medical Sciences and Peking Union Medical College, Beijing, China

**Keywords:** Burnout, Physician, Resident, Mental health, Network analysis, Sleep, Anxiety, Self-efficacy, Depression

## Abstract

**Background:**

Burnout affects the physical and mental health of residents. This study aims to investigate the association between burnout and health across biological, psychological, and social domains among Chinese residents.

**Method:**

A cross-sectional study was conducted from July 2022 to June 2024 at Peking Union Medical College Hospital in China. A total of 274 resident physicians were recruited, with 207 providing valid responses. Burnout was assessed using the Maslach Burnout Inventory-Human Services Survey (MBI-HSS), while general health was measured using the Union Physio-Psycho-Social Assessment Questionnaire (UPPSAQ-70). Self-efficacy and subjective incompetence were also evaluated. Network analysis was applied to identify the centrality of various health factors in relation to burnout.

**Results:**

The overall prevalence of burnout was 74.4% (95% CI: 68.5-79.9%). Emotional exhaustion and depersonalization were significantly higher in male physicians compared to females, while younger physicians (aged ≤ 30 years) reported higher burnout in personal achievement. Network analysis identified strong connections between burnout dimensions (emotional exhaustion and depersonalization) and mental health symptoms such as anxiety, fatigue, and depression. Sleep, anxiety, and self-efficacy were found to be central nodes in the network, indicating their crucial role in resident physicians’ well-being.

**Conclusions:**

This study found a high prevalence of burnout among resident physicians, with significant links between burnout and mental health conditions. Anxiety and fatigue emerge as core symptoms in the burnout network, suggesting that interventions should address these factors. Additionally, self-efficacy plays a crucial role in burnout.

## Background

There is growing concern about the well-being of physicians, and the impact of poor well-being on both the individuals themselves and the healthcare system [1]. Over the past 15 years, the term “burnout” has gained prominence as a descriptor of poor well-being among physicians. Burnout is defined in the 11th Revision of the International Classification of Diseases (ICD-11) as a multi-dimensional syndrome encompassing emotional exhaustion, depersonalization, and diminished feelings of personal accomplishment [2]. The prevalence of burnout among physicians varies depending on the criteria used, with most studies reporting rates greater than 50% [1], while some report an incidence ranging from 0 to 80.5% [3–5]. Physician burnout is associated with increased medical errors, lower patient satisfaction, prolonged post-discharge recovery times, and reduced professional work effort [6–8].

Continuous measures of depression and burnout symptoms are highly correlated, indicating significant overlap between these two constructs [9]. While burnout is defined as an occupational phenomenon, conditions such as depression, anxiety, and insomnia are also major concerns in the medical field. Studies suggest that approximately one-third to one-half of resident physicians experience symptoms of depression and anxiety [10, 11]. Moreover, residents experiencing burnout are more likely to report higher rates of depression and anxiety, which can exacerbate feelings of hopelessness and disengagement. In recent years, there has been increasing recognition of the interconnections between burnout and other mental health issues [12, 13].

Network analysis (NA), a symptom-based approach, has proven effective in exploring these complex relationships. Addressing the interplay between burnout and these co-occurring mental health conditions is essential for developing a comprehensive understanding of resident well-being. A network analysis study has suggested that burnout extends beyond workplace issues, with strong connections to depression, anxiety, and low quality of life [14]. However, the tools used in this study were relatively simple, relying on a two-item questionnaire to assess burnout, and did not consider the broader physiological, psychological, and social health status.

Despite progress, significant gaps remain in our understanding of how burnout and mental health issues interact among residents. While some interventions have shown promise in reducing burnout, they often do not address the full spectrum of mental health challenges faced by residents. By gaining a deeper understanding of the complex interactions between these factors, we can develop more effective strategies to protect the health and well-being of residents and, by extension, improve the overall healthcare system. Therefore, this study aims to investigate the association between burnout and health across biological, psychological, and social domains among Chinese residents.

## Methods

### Study design and participants

This cross-sectional study was conducted at Peking Union Medical College Hospital in China. The study was conducted from July 2022 to June 2024, with participants recruited through convenience sampling from 10 wards at the hospital. All participants provided electronic informed consent. The study adhered to the Strengthening the Reporting of Observational Studies in Epidemiology (STROBE) Statement for reporting observational studies. Patients and the public were not involved in the design, conduct, reporting, or dissemination of this research. The inclusion criteria were: (1) aged ≥ 18 years, (2) resident physicians working on the ward, regardless of their specialty, and (3) agreed to complete the questionnaires. The exclusion criteria for the valid responses were as follows: total completion time < 300 s (considered to be at or above the 95th percentile for general population questionnaire completion times) or selecting the same response options across the entire survey.

### Assessment

The 22-item Maslach Burnout Inventory-Human Services Survey (MBI-HSS) was used to measure burnout [15], which encompasses three domains with corresponding subscales: emotional exhaustion (EE), depersonalization (DP), and personal achievement (PA). Participants were asked to respond using a seven-point Likert scale ranging from a score of 0–6 (0 = never, 6 = everyday). Scores of EE ≥ 27, DP ≥ 10, and PA ≤ 33 were considered indicative of high burnout. The overall burnout threshold was defined by EE ≥ 27 or DP ≥ 10 [5].

The Union Physio-Psycho-Social Assessment Questionnaire (UPPSAQ-70) was used to measure general health factors. The questionnaire was initially developed to address the assessment of the comprehensive physio-psycho-social health status of patients in general hospitals [16]. It includes 8 factors: depression, anxiety and fatigue, sleep, physical discomfort, sexual function, happiness and satisfaction, hypochondria, and social anxiety. A positive result was defined by a factor score ≥ 1 or a total score ≥ 65. The Cronbach’s *α* of each factor and the total score are between 0.954 and 0.956 [16].

Subjective incompetence and self-efficacy were evaluated using the Subjective Incompetence Scale (SIS) and the General Self-Efficacy Scale (GSES), respectively. The SIS, a 12-item scale, assesses demoralization, with higher total scores indicating higher subjective incompetence (range 0–36), and a reliability coefficient of 0.90 [17]. The GSES, a 10-item unidimensional scale, measures belief in one’s ability to handle novel or difficult situations. Responses were made on a 4-point scale, with the total score ranging from 10 to 40. Cronbach’s alpha for this scale was between 0.82 and 0.93 [18].

Socioeconomic data, including age, sex, years of education, employment status, family income, and marital status, were collected during enrollment.

### Statistical analysis

Continuous variables are presented as the mean ± standard deviation. Categorical variables are presented as numbers (%). Group differences in continuous variables were analyzed using Student’s t-test or one-way analysis of variance (ANOVA) with Scheffé’s post hoc test, as appropriate.

Associations between burnout and sociodemographic factors were examined through univariate analysis. Categorical variables were analyzed using the Wilcoxon signed-rank test or Kruskal-Wallis H test, while continuous variables were assessed using Spearman’s correlation coefficients. p-values after Bonferroni correction were reported for multiple comparisons. Analysis of covariance (ANCOVA) was employed to account for covariates and their interactions.

The network analysis included nodes representing age, the eight UPPSAQ-70 factors, the SIS, the GSES, and the three MBI-HSS subscales. Data were normalized, and the value of PA was inverted. The edges were the partial correlations between pairs of nodes after controlling for other variables in the network. Thicker edges indicate stronger associations, with red edges representing negative correlations and blue edges representing positive correlations. The network structure was visualized using the Fruchterman-Reingold algorithm, with central nodes placed closer to the center of the network. Network centrality was assessed using strength, closeness, and betweenness. Strength takes the sum of absolute edge weights connected to each node. Closeness takes the inverse of the sum of distances from one node to all other nodes. Betweenness quantifies how often one node is in the shortest path between another node. Multicollinearity was evaluated through regression diagnostics and the Variance Inflation Factor (VIF) [19].

Statistical analyses were conducted using Statistical Product and Service Solutions v20.0 (SPSS Inc., IBM Company, Chicago, IL, USA) and R software (bootnet and qgraph package) [20, 21].All the statistical tests were two-tailed, with significance set at *p* < 0.05.

## Results

A total of 274 participants were included in this study, and 196 were included after excluding unqualified questionnaires. The average age of the participants was 31.86 ± 7.02 years, 50 (25.5%) of whom were male. The participants came from 10 wards, with the number of participants in each ward ranging from 22 (Gynecology) to 33 (Cardiology). Table 1 shows more detailed information on the demographic characteristics of the study sample.

**Table 1:** Sample characteristics of resident physicians

Characteristic	N	MBI-HSS, Median (IQR)			UPPSAQ-70, Median (IQR)								SIS, Median (IQR)	GSES, Median (IQR)
		Emotional exhaustion	Depersonalization	Personal achievement	Depression	Anxiety and fatigue	Sleep	Physical discomfort	Sexual function	Happiness and satisfaction	Hypochondria	Social anxiety		
Sex														
Female	146	17.0(9.0–29.0)	6.0(2.8–10.0)	32.0(22.0–40.0)	0.6(0.1–0.9)	0.6 (0.3–1.1)	0.4(0.1–0.8)	0(0–0.7)	2.0(1.6–2.0)	1.0(0.5–1.6)	0.2(0.6–0.9)	0.7(0.3–1.2)	14.0(11.0–16.0)	2.8(2.3–3.2)
Male	50	24.0(14.0–37.0)	9.0(4.5–17.0)	32.0(22.0–38.0)	0.7(0.4–1.0)	0.8(0.4–1.1)	0.6(0.4–1.0)	0(0–0.7)	1.8(1.2–2.0)	1.3(1.0–1.7)	0.3(0.0–0.8)	0.8(0.5–1.2)	14.0(8.5–18.0)	2.8(2.5–3.1)
Age, years														
≤ 30	114	21.0(11.0–35.0)	7.0(4.0–13.0)	35.0(24.0–40.0)	0.6(0.2–0.9)	0.7(0.3–1.1)	0.4(0.1–0.8)	0(0–0)	1.9(1.5–2)	1.0(0.6–1.6)	0.3(0.1–0.8)	0.8(0.3–1.2)	14.0(8.3–16.0)	2.8(2.4–3.2)
> 30	82	17.0(9.0–30.0)	6.0(3.0–10.0)	28.0(19.0–36.0)	0.6(0.2–0.9)	0.7(0.4–1.1)	0.5(0.3–0.9)	0.3(0–1.0)	2.0(1.7–2.0)	1.1(0.7–1.8)	0.5(0–1.3)	0.7(0.3–1.0)	14.0(12.0–17.0)	2.8(2.3–3.1)
Department														
Internal	135	19.5(11.0–34.8)	7.0(4.0–12.0)	30.0(22.0–38.0)	0.6(0.2–0.9)	0.6(0.3–1.05)	0.4(0.1–0.8)	0(0–0.7)	2.0(1.6–2.0)	1.0(0.6–1.6)	0.7(0.3–1.2)	0.4(0.1–1.1)	14.0(10.0–16.0)	2.8(2.3–3.1)
Cardiology	33	16.0(8.0–33.0)	6.0(2.0–12.0)	33.0(25.0–40.0)	0.6(0.1–0.9)	0.6(0.2–1.1)	0.4(0.1–0.6)	0(0–0.7)	2.0(1.6–2.0)	1.0(0.7–1.6)	0.4(0–0.9)	0.7(0.3–1)	14.0(12.0–15.5)	2.9(2.5–3.1)
Neurology	29	16.0(12.5–27.0)	7.0(3.5–12.0)	22.0(19.5–27.5)	0.6(0.3–1.0)	0.7(0.5–1.1)	0.4(0.3–0.6)	0(0–0.4)	2.0(1.6–2.1)	1.0(0.7–1.6)	0.3(0.1–0.9)	1.0(0.5–1.2)	12.0(0–14.0)	2.7(2.6–2.9)
Immunology	25	20.0(9.0–29.5)	6.0(1.5–10.0)	35.0(26.0–39.0)	0.3(0.1–0.9)	0.6(0.3–0.9)	0.4(0.2–0.8)	0(0–0.9)	1.9(1.6–2.2)	1.2(0.6–1.6)	0.6(0.1–1.1)	0.8(0.4–1.2)	14.0(11.0–17.0)	2.7(2.2–3.2)
Pulmonology	18	24.0(8.8–40.0)	9.5(4.8–17.5)	36.0(22.5–40.3)	0.6(0.2–0.9)	0.7(0.4–1.0)	0.4(0.1–0.7)	0(0–0.4)	1.8(1.3–2.0)	1.1(0.7–1.6)	0.7(0.3–1.4)	0.7(0.5–1.9)	12.5(0–15.8)	3.0(2.0–3.2)
Endocrinology	15	24.0(18.0–42.0)	7.0(5.0–15.0)	29.0(24.0–35.0)	0.9(0.5–1.3)	1.1(0.6–1.6)	1.0(0.4–1.5)	0.9(0–1.6)	2.0(1.6–2.4)	1.3(0.6–1.8)	1.0(0.3–1.5)	1.0(0.6–1.2)	15.0(12.0–17.0)	2.3(1.9–2.9)
Gastroenterology	15	17.0(9.0–34.0)	8.0(6.0–15.0)	25.0(20.0–37.0)	0.6(0.2–0.7)	0.5(0.2–0.9)	0.4(0.1–0.7)	0(0–0.7)	1.8(1.4–2.0)	0.8(0.2–1.3)	0.2(0–1.0)	0.3(0–0.8)	12.0(9.0–15.0)	3.0(2.8–3.4)
Surgery	61	19.0(8.0–27.0)	6.0(2.0–11.0)	36.0(22.0–40.0)	0.7(0.2–1.0)	0.8(0.3–1.2)	0.5(0.1–0.9)	0(0–0.7)	2.0(1.6–2.0)	1.3(0.6–1.7)	0.8(0.5–1.0)	0.3(0–1.0)	14.0(12.0–18.0)	3.0(2.6–3.5)
General Surgery	21	20.0(7.5–31.5)	6.0(2.0–9.5)	37.0(24.5–40.0)	0.7(0.2–1.0)	0.6(0.5–1.1)	0.5(0.1–0.8)	0(0–0.7)	2.0(1.7–2.0)	1.1(0.9–1.8)	0.3(0–0.9)	0.8(0.5–1.1)	14.0(12.5–15.5)	2.9(2.6–3.1)
Urology	16	11.0(1.3–28.3)	3.0(0–11.8)	33.0(21.0–42.8)	0.6(0.1–1.1)	0.8(0.1–1.1)	0.4(0–0.9)	0(0–1.0)	2.0(1.7–2.0)	1.2(0.4–1.6)	0.2(0–0.7)	0.6(0.1–1.0)	15.0(10.5–19.8)	3.7(2.4–4.0)
Orthopedics	14	23.0(8.3–37.0)	7.5(3.3–17.3)	39.0(26.8–41.0)	0.7(0.3–1.1)	0.8(0.3–1.4)	0.5(0.4–1.0)	0(0–0.7)	1.8(1.5–2)	1.3(0.5–1.6)	0.3(0–1.0)	1.0(0.6–1.2)	15.5(12.3–17.3)	2.9(2.6–3.1)
Gynecology	22	19.0(15.8–25.5)	7.5(4.8–10.3)	20.5(18.8–38.0)	0.6(0.2–1.1)	1.0(0.5–1.7)	0.6(0.1–1.1)	0(0–0.8)	2.0(1.5–2.3)	1.2(0.1–1.9)	0.7(0.1–1.2)	1.0(0.6–1.2)	13.0(0–17.3)	2.4(2.0–3.1)
Total	196	19.0(9.0–32.0)	7.0(3.0–12.0)	32.0(22.0–40.0)	0.6(0.2–0.9)	0.7(0.3–1.1)	0.4(0.1–0.8)	0(0–0.7)	2.0(1.6–2.0)	1.1(0.6–1.6)	0.3(0–1.0)	0.7(0.3–1.2)	14.0(11.0–16.0)	2.8(2.4–3.2)
Positive rate, n(%)					49(23.7)	73(35.3)	37(17.9)	38(18.4)	191(92.3)	122(58.9)	55(26.6)	83(40.1)		

MBI-HSS—Maslach Burnout Inventory-Human Services Survey, IQR—Inter-quartile range, UPPSAQ-70—Union Physio-Psycho-Social Assessment Questionnaire, SIS—Subjective Incompetence Scale, GSES—General Self-Efficacy Scale

The scores of the MBI-HSS were 19.0 (9.0–32.0) for EE, 7.0 (3.0–12.0) for DP and 32.0 (22.0–40.0) for PA. The prevalence rate of overall high burnout was: 74.4% (95% confidence interval [CI]: 68.5-79.9%). There were significant differences between the two sexes in the scores of EE (*p* = 0.011 < 0.05) and DP (*p* = 0.002 < 0.05). No significant difference between the two sexes in the scores of PA (*p* = 0.527 > 0.05). There were significant differences in PA scores between different age groups (*p* = 0.001 < 0.01), but no significant differences in other scores (EE: *p* = 0.242 > 0.05; DP: *p* = 0.106 > 0.05) between different age groups. There were no significant differences in the three scale scores between the Internal and the Surgery departments (EE: *p* = 0.147 > 0.05; DP: *p* = 0.282 > 0.05; PA: *p* = 0.121 > 0.05).

**Fig. 1:**
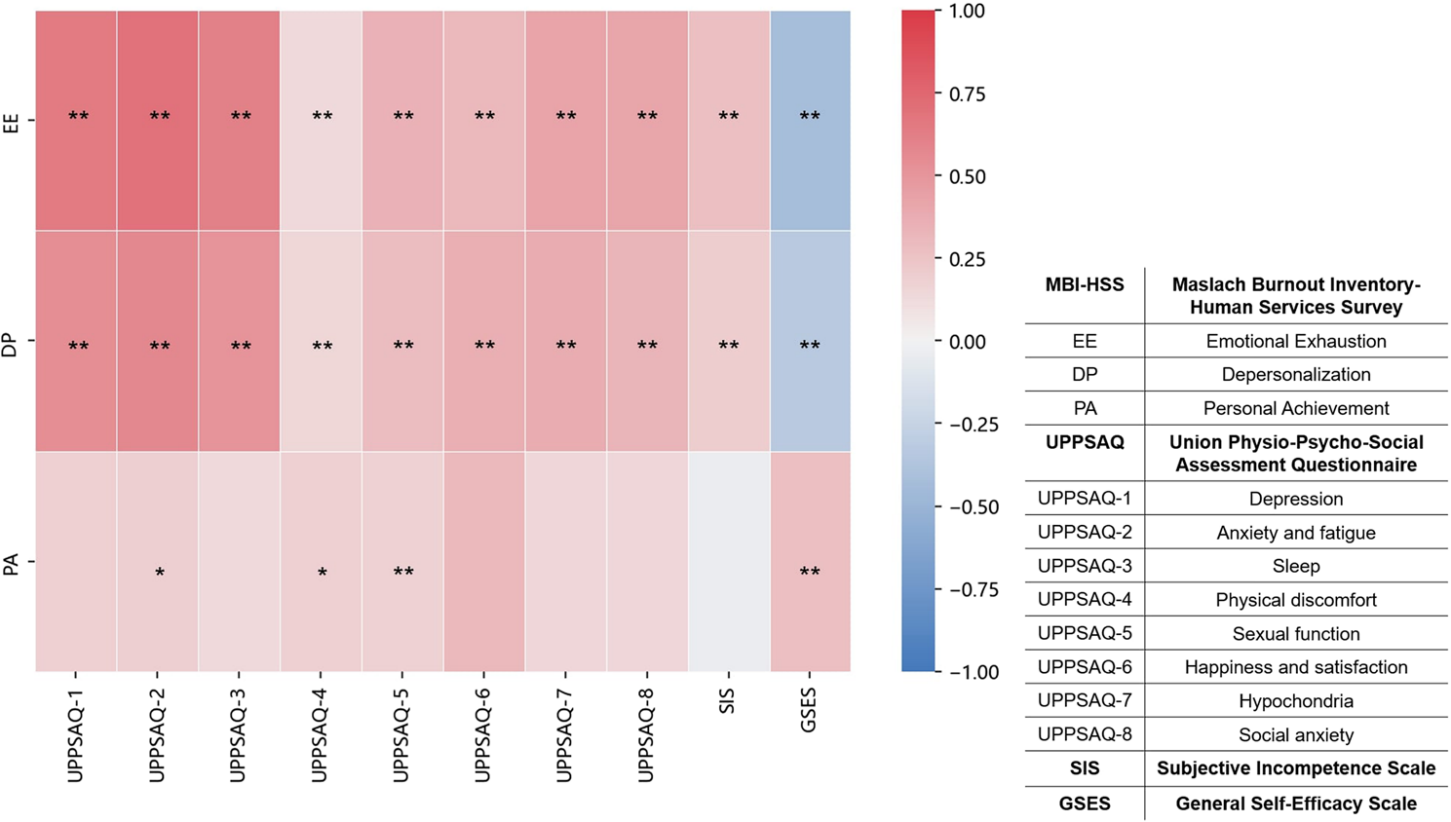
The correlations between the variables. *p < 0.05, **p < 0.01

EE was significantly correlated with all dimensions of the UPPSAQ-70 (*r* = 0.32–0.68, *p* < 0.01), except for sexual function, and was positively correlated with the SIS (*r* = 0.27, *p* < 0.01) and negatively correlated with the GSES (*r* = -0.36, *p* < 0.01). DP was significantly correlated with all dimensions of the UPPSAQ-70 (*r* = 0.26–0.55, *p* < 0.01), except for sexual functioning, and was positively correlated with the SIS (*r* = 0.24, *p* < 0.01) and negatively correlated with the GSES (*r* = -0.29, *p* < 0.01). PA was significantly negatively correlated with age (*r* = -0.29, *p* < 0.01) and happiness and satisfaction (*r* = -0.31, *p* < 0.01), and positively correlated with the GSES (*r* = 0.28, *p* < 0.01), but showed no significant correlation with other variables (*p* > 0.05). See Fig. 1 for details.

**Fig. 2:**
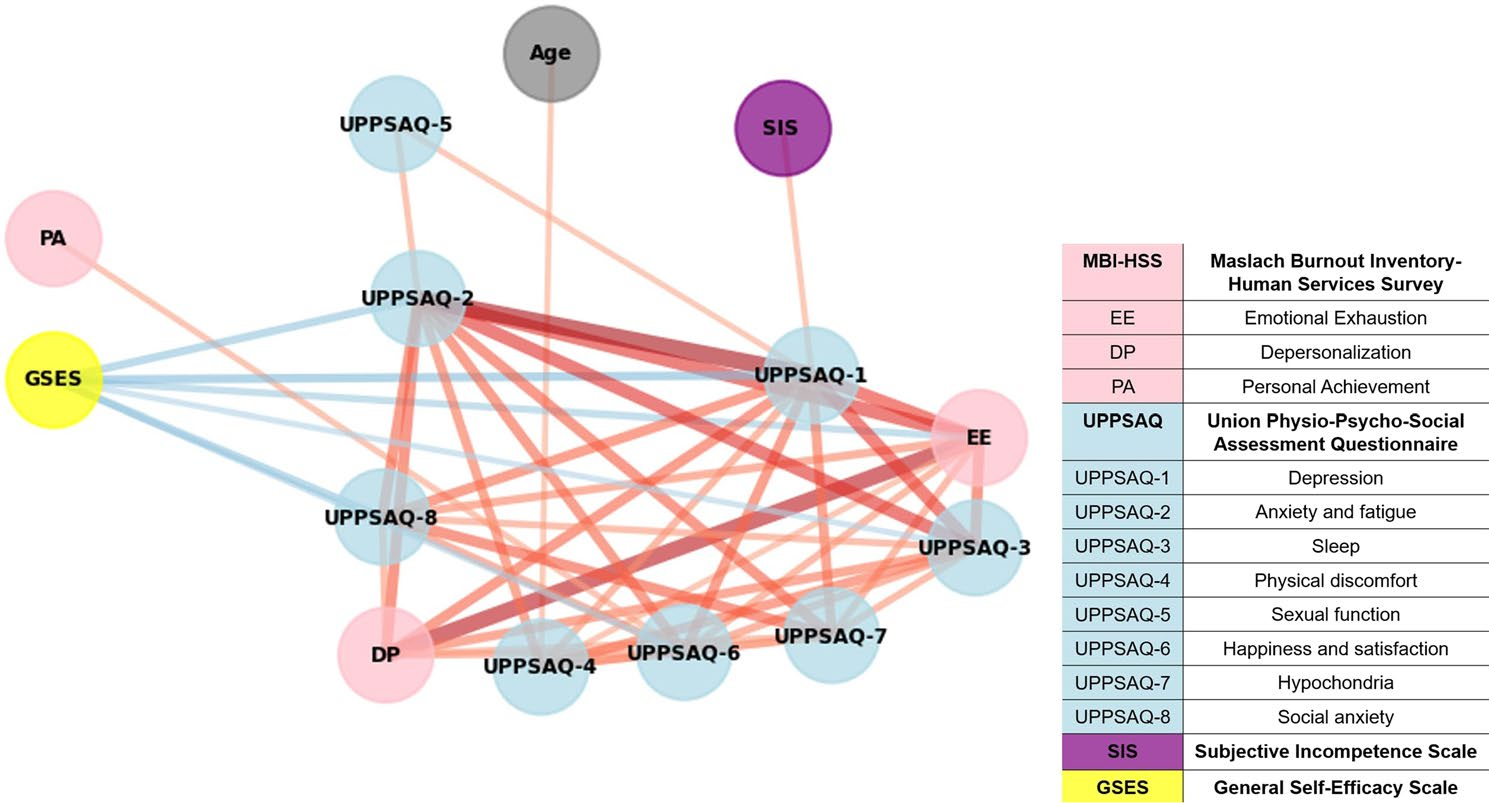
The network structure. When the threshold value of the weight is 0.3, a total of 45 edges were displayed in the network analysis. Thicker edges indicate stronger associations, with red edges representing negative associations and blue edges denoting positive associations

The network analysis was represented in Fig. 2. When the threshold value of the weight is 0.3, a total of 45 edges were displayed in the network analysis. All edges within the network were positive except the ones associated with GSES. Among the factors in the UPPSAQ-70, there was a strong link between UPPSAQ-1 (depression), UPPSAQ-2 (anxiety and fatigue) and UPPSAQ-3 (sleep). Among factors in MBI-HSS, there was a strong link between MBI-HSS-EE (emotional exhaustion) and MBI-HSS-DP (depersonalization), while no link was represented with MBI-HSS-PA (personal achievement). The strongest connection (i.e., the highest edge weight) between UPPSAQ-70 and MBI-HSS factors was observed between UPPSAQ-2 (anxiety and fatigue) and EE. In the network, MBI-HSS-PA was only connected to happiness and satisfaction, SIS was only linked to depression, and GSES was associated with EE in the MBI-HSS, as well as with anxiety and vulnerability, depression, sleep, and happiness and satisfaction in the UPPSAQ-70.

**Fig. 3:**
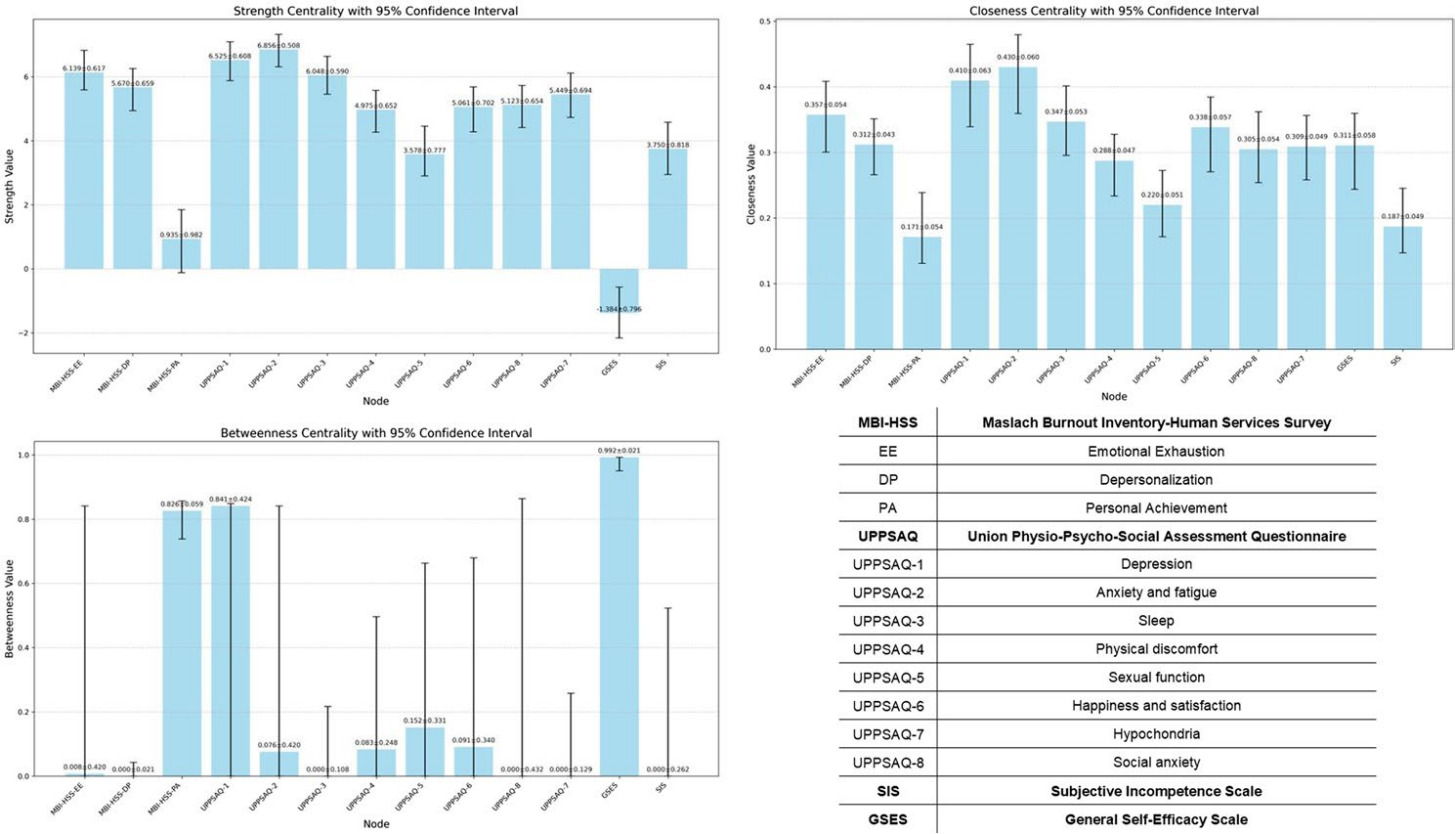
The degree centrality of nodes and the bootstrap results

Figure 3 display the strength, closeness, and betweenness of nodes and the bootstrap results. UPPSAQ-2 (anxiety and fatigue) had the highest strength centrality and closeness centrality across the entire network. Other central symptoms included UPPSAQ-1 (depression) and MBI-HSS-EE (emotional exhaustion). GSES, UPPSAQ-1 (depression), and HBI-HISS-PA had higher betweenness centrality.

## Discussions

This study included 196 physicians from 10 departments. The scores on the MBI-HSS were 19.0 (9.0–32.0) for EE, 7.0 (3.0–12.0) for DP, and 32.0 (22.0–40.0) for PA. Male physicians had higher scores in the dimensions of emotional exhaustion and depersonalization. No significant differences were found between internal and surgery departments. Burnout was closely associated with physical-psychological symptoms and had a strong relationship with subjective incompetence and self-efficacy. This study also established a network relationship between physical-psychological symptoms and burnout, where anxiety and fatigue, depression, and emotional exhaustion emerged as core symptoms within the network.

Previous studies showed that the prevalence of burnout symptoms among Chinese doctors ranged from 66.5–87.8% [22, 23], and a multicenter study in China reported a prevalence of physician burnout of 60.8% [24]. In a global meta-analysis, the overall prevalence rate of occupational burnout was 67% [5]。The burnout prevalence rate in this study was 74.4% (95% CI: 68.5-79.9%), which is similar to the overall rate reported in the aforementioned study. Many cross-sectional studies have found an independent relationship between occupational burnout and the gender and age of physicians [25]。Some studies in the United States and Finland have found that women experience higher levels of occupational burnout than men [26–28]. However, studies on occupational burnout among Chinese physicians have found that male physicians experience burnout at equal or higher levels than female physicians [14, 23, 24, 29, 30], which may be related to different cultural backgrounds. Several studies have found that younger physicians are more likely to experience occupational burnout than older physicians [25, 29], And it is often concentrated in the dimension of personal achievement. One possible explanation is that employees tend to categorize demands as challenges, which are positively correlated with engagement [31]. Therefore, although older physicians may face more challenging tasks, which can lead to overload and loss of enthusiasm, these challenges may also enhance their sense of achievement.

In some studies, approximately 25 to 30% of physicians screen positive for depression [32], to which the prevalence (23.7%) in this study is similar. Researchers generally consider depression to be the psychological symptom most closely related to occupational burnout. There has been an ongoing debate regarding the overlap between burnout and depression constructs. Specifically, recent studies have increasingly highlighted a substantial overlap between burnout and depression, suggesting that burnout might be considered as one dimension of depression rather than a distinct psychological phenomenon [33]. However, in this study, depression and burnout exhibited distinct characteristics within the network of physical-psychological symptoms and burnout among resident physicians, suggesting that they are two separate concepts. First, in the correlation analysis, depression showed significant associations with emotional exhaustion and depersonalization, but not with personal accomplishment, indicating that the personal accomplishment dimension serves as an important differentiator between depression and burnout. The relationship between self-efficacy and personal accomplishment in burnout is more closely related, with the ICD-11 describing this dimension as “reduced professional efficacy”. Furthermore, in the network analysis, anxiety and fatigue exhibited higher centrality than depressive symptoms, suggesting that while depression is important in the network of physical-psychological symptoms and burnout, it is not all-encompassing. Viewing burnout solely as depressive symptoms manifesting in professional performance overlooks the connections between burnout and other physical-psychological issues faced by resident physicians.

In this study, 35.27% of physicians reported anxiety and fatigue, while other studies have reported anxiety disorder prevalence rates ranging from 27.2 to 51.6% [34–36]. In the network of physical-psychological symptoms and burnout in this study, anxiety and fatigue exhibited the highest centrality, and emotional exhaustion from burnout was found to have a stronger positive correlation with anxiety and fatigue. In another network analysis, although emotional exhaustion was closely associated with the symptom cluster of depression, it showed the strongest correlation with the “Fatigue” item from the PHQ [14]. This suggests that anxiety and fatigue require more attention in the physical and mental health of resident physicians. This suggests a close interaction between the psychological and physical aspects of occupational burnout, such as emotional exhaustion and fatigue, providing important targets for intervention.

Self-efficacy is an important key variable in the physical-psychological-professional burnout network of resident physicians. In this study, self-efficacy showed significant correlations with all three dimensions of burnout, as well as with all dimensions of the UPPSAQ-70, except for sexual functioning. Other studies have also identified a close association between self-efficacy, burnout, and the well-being of resident physicians [37, 38]. Therefore, in the process of intervening in burnout, the assessment of self-efficacy should not be overlooked. Reducing working hours has long been considered an important strategy for mitigating burnout. However, some studies have pointed out that restrictions on working hours may lead to a lack of confidence in the ability to work independently among surgical residents [39]. While addressing burnout, it is important to ensure that self-efficacy is not compromised.

The Self-Determination Theory posits that the satisfaction of basic psychological needs—autonomy, competence, and relatedness—is essential for psychological wellbeing and optimal functioning [40]. Subjective incompetence reflects a loss of autonomy. In the correlation analysis of this study, subjective incompetence was significantly correlated with Emotional Exhaustion and Depersonalization, but showed no significant association with Personal Accomplishment. In the network analysis, subjective incompetence was only linked to depression. This further suggests that burnout and depression involve distinct psychological components.

This study reported that 92.3% of resident physicians exhibited positive symptoms related to sexual dysfunction. Research on sexual function and its associated factors among resident physicians is currently limited, possibly due to excessive workload and long working hours, which affect the ability to establish and maintain intimate relationships. Although this issue does not have a direct association with occupational burnout, it warrants further attention.

This study also has several limitations. First, it did not include objective factors such as work stress and working hours, which can influence occupational burnout. Future research could design studies to incorporate these factors and improve the network. Second, the sample size in this study was limited, and it was drawn from only one hospital. Future studies with larger sample sizes and multi-center designs could further validate these findings. Thirdly, while this study identified some potential targets for improving occupational burnout, future research could design comprehensive interventions targeting these factors and conduct prospective controlled trials to clarify the significance of these targets. Finally, the assessment of depression was based solely on self-report questionnaires, without confirmation through clinical interviews or medical records. This limits the ability to draw firm conclusions about diagnostic status and highlights the need for future studies to incorporate multiple sources of clinical data.

## Conclusion

This study confirms the high prevalence of burnout among Chinese resident physicians and demonstrates its strong association with physical and psychological symptoms. Anxiety and fatigue emerge as core symptoms in the burnout network, suggesting that interventions should address these factors. Additionally, self-efficacy plays a crucial role in burnout. Future research should explore comprehensive intervention strategies targeting burnout and its associated mental health issues.

## Data Availability

The datasets used and/or analyzed during the current study are available from the corresponding author on reasonable request.

## Abbreviations

ANCOVA: Analysis of covariance
CI: Confidence interval
DP: depersonalization
EE: Emotional exhaustion
GSES: General self-efficacy scale
ICD: 11-International Classification of Diseases, 11th Revision
IQR: Inter-quartile range
MBI: HSS-Maslach Burnout Inventory-Human Services Survey
NA: Network analysis
PA: Personal achievement
PHQ: Patient health questionnaire
SIS: Subjective incompetence scale
STROBE: Strengthening the reporting of observational studies in epidemiology
UPPSAQ: 70-Union Physio-Psycho-Social Assessment Questionnaire

## References

[1] Guille C, Sen S. Burnout, depression, and diminished Well-Being among physicians. NEW ENGL J MED. 2024;391(16):1519–27. 39442042 10.1056/NEJMra2302878

[2] ICD-11: International classification of diseases (11th revision); 2022.

[3] Pantenburg B, Luppa M, Konig HH, Riedel-Heller SG. Burnout among young physicians and its association with physicians’ wishes to leave: results of a survey in saxony, Germany. J OCCUP MED TOXICOL. 2016;11:2. 26807138 10.1186/s12995-016-0091-z PMC4724157

[4] Kansoun Z, Boyer L, Hodgkinson M, Villes V, Lancon C, Fond G. Burnout in French physicians: A systematic review and meta-analysis. J AFFECT DISORDERS. 2019;246:132–47. 30580199 10.1016/j.jad.2018.12.056

[5] Rotenstein LS, Torre M, Ramos MA, Rosales RC, Guille C, Sen S, Mata DA. Prevalence of burnout among physicians: A systematic review. JAMA-J AM MED ASSOC. 2018;320(11):1131–50. 10.1001/jama.2018.12777 PMC6233645 30326495

[6] West CP, Tan AD, Habermann TM, Sloan JA, Shanafelt TD. Association of resident fatigue and distress with perceived medical errors. JAMA-J AM MED ASSOC. 2009;302(12):1294–300. 10.1001/jama.2009.1389 19773564

[7] Shanafelt TD, Mungo M, Schmitgen J, Storz KA, Reeves D, Hayes SN, Sloan JA, Swensen SJ, Buskirk SJ. Longitudinal study evaluating the association between physician burnout and changes in professional work effort. MAYO CLIN PROC. 2016;91(4):422–31. 27046522 10.1016/j.mayocp.2016.02.001

[8] Vaquero-Cepeda P, Pujol-de CA, Catala-Lopez F. Association between physician burnout and patient safety: study protocol for an overview of systematic reviews and meta-analyses. BMJ OPEN. 2024;14(11):e89687. 10.1136/bmjopen-2024-089687 PMC11590821 39578025

[9] Rotenstein LS, Zhao Z, Mata DA, Guille C, Sen S. Substantial overlap between factors predicting symptoms of depression and burnout among medical interns. J GEN INTERN MED. 2021;36(1):240–2. 32026254 10.1007/s11606-020-05664-x PMC7859012

[10] Mata DA, Ramos MA, Bansal N, Khan R, Guille C, Di Angelantonio E, Sen S. Prevalence of depression and depressive symptoms among resident physicians: A systematic review and Meta-analysis. JAMA-J AM MED ASSOC. 2015;314(22):2373–83. 10.1001/jama.2015.15845 PMC4866499 26647259

[11] Shafiee A, Teymouri AM, Seighali N, Amini MJ, Hajishah H, Arabazadeh BR, Akhoundi A, Beiky M, Sarvipour N, Maleki S, et al. The prevalence of depression, anxiety, and sleep disturbances among medical students and resident physicians in iran: A systematic review and meta-analysis. PLoS ONE. 2024;19(8):e307117. 10.1371/journal.pone.0307117 PMC11343466 39178292

[12] Williford ML, Scarlet S, Meyers MO, Luckett DJ, Fine JP, Goettler CE, Green JM, Clancy TV, Hildreth AN, Meltzer-Brody SE, et al. Multiple-Institution comparison of resident and faculty perceptions of burnout and depression during surgical training. JAMA SURG. 2018;153(8):705–11. 29800976 10.1001/jamasurg.2018.0974 PMC6584717

[13] Dyrbye LN, Shanafelt TD. Physician burnout: a potential threat to successful health care reform. JAMA-J AM MED ASSOC. 2011;305(19):2009–10. 10.1001/jama.2011.652 21586718

[14] Li Z, Wu M, Zhang X, Yan K, Wang X, Xu H, Li P, Liu Y, Deng Q, Li X, et al. Interrelationships of stress, burnout, anxiety, depression, quality of life and suicidality among Chinese residents under standardized residency training: a network analysis. ANN MED. 2024;56(1):2433030. 39610267 10.1080/07853890.2024.2433030 PMC11610237

[15] Dyrbye LN, West CP, Leep HA, Johnson P, Cipriano P, Peterson C, Beatty D, Major-Elechi B, Shanafelt T. Relationship between burnout and professional behaviors and beliefs among US nurses. J OCCUP ENVIRON MED. 2020;62(11):959–64. 32868601 10.1097/JOM.0000000000002014

[16] Duan YP, Wei J, Hong X, Cao JY, Shi LL, Zhao XH, Ke MY, Zhu YJ, Li SW, Jiang J, et al. [Psychometric evaluation of the union Physio-Psycho-Social assessment questionnaire]. Zhongguo Yi Xue Ke Xue Yuan Xue Bao. 2019;41(5):615–21. 31699191 10.3881/j.issn.1000-503X.10960

[17] Cockram CA, Doros G, de Figueiredo JM. Diagnosis and measurement of subjective incompetence: the clinical hallmark of demoralization. PSYCHOTHER PSYCHOSOM. 2009;78(6):342–5. 19713728 10.1159/000235737

[18] Barlow JH, Williams B, Wright C. The generalized Self-Efficacy scale in people with arthritis. Arthritis Care Res. 1996;9(3):189–96. 8971228 10.1002/1529-0131(199606)9:3<189::aid-anr1790090307>3.0.co;2-#

[19] Hart PD. Partial relationships between health and fitness measures in adults: A network analysis. Am J Public Health Res. 2022;4(10):147–53.

[20] Epskamp S, Borsboom D, Fried EI. Estimating psychological networks and their accuracy: A tutorial paper. BEHAV RES METHODS. 2018;50(1):195–212. 28342071 10.3758/s13428-017-0862-1 PMC5809547

[21] Epskamp S, Cramer AOJ, Waldorp LJ, Schmittmann VD, Borsboom D. Qgraph: network visualizations of relationships in psychometric data. J STAT SOFTW. 2012;4(48):1–18.

[22] Lo D, Wu F, Chan M, Chu R, Li D. A systematic review of burnout among Doctors in china: a cultural perspective. Asia Pac Fam Med. 2018;17:3. 29449785 10.1186/s12930-018-0040-3 PMC5806482

[23] Feng S, Li X, Huang Z, Jiang C, Cheng X, Ma Y, Zhang F, Meng X. The relationship between burnout and sense of school belonging among the resident physicians in the standardization training in China. MED EDUC ONLINE. 2024;29(1):2343515. 38660991 10.1080/10872981.2024.2343515 PMC11047212

[24] Xiao Y, Dong D, Zhang H, Chen P, Li X, Tian Z, Jing Z, Zhang S. Burnout and Well-Being among medical professionals in china: A National Cross-Sectional study. FRONT PUBLIC HEALTH. 2021;9:761706. 35111713 10.3389/fpubh.2021.761706 PMC8801677

[25] West CP, Dyrbye LN, Shanafelt TD. Physician burnout: contributors, consequences and solutions. J INTERN MED. 2018;283(6):516–29. 29505159 10.1111/joim.12752

[26] McMurray JE, Linzer M, Konrad TR, Douglas J, Shugerman R, Nelson K. The work lives of women physicians results from the physician work life study. The SGIM career satisfaction study group. J GEN INTERN MED. 2000;15(6):372–80. 10886471 10.1111/j.1525-1497.2000.im9908009.x PMC1495474

[27] West CP, Shanafelt TD, Kolars JC. Quality of life, burnout, educational debt, and medical knowledge among internal medicine residents. JAMA-J AM MED ASSOC. 2011;306(9):952–60. 10.1001/jama.2011.1247 21900135

[28] Shanafelt TD, Oreskovich MR, Dyrbye LN, Satele DV, Hanks JB, Sloan JA, Balch CM. Avoiding burnout: the personal health habits and wellness practices of US surgeons. ANN SURG. 2012;255(4):625–33. 22388107 10.1097/SLA.0b013e31824b2fa0

[29] Huang L, Caspari JH, Sun X, Thai J, Li Y, Chen FZ, Zhao XD. Risk and protective factors for burnout among physicians from standardized residency training programs in shanghai: a cross-sectional study. BMC HEALTH SERV RES. 2020;20(1):965. 33087121 10.1186/s12913-020-05816-z PMC7576715

[30] Wang Z, Xie Z, Dai J, Zhang L, Huang Y, Chen B. Physician burnout and its associated factors: a cross-sectional study in Shanghai. J OCCUP HEALTH. 2014;56(1):73–83. 24430838 10.1539/joh.13-0108-oa

[31] Chirico F. Job stress models for predicting burnout syndrome: a review. ANN I SUPER SANITA. 2016;52(3):443–56. 10.4415/ANN_16_03_17 27698304

[32] Sen S, Kranzler HR, Krystal JH, Speller H, Chan G, Gelernter J, Guille C. A prospective cohort study investigating factors associated with depression during medical internship. Arch Gen Psychiatry. 2010;67(6):557–65. 20368500 10.1001/archgenpsychiatry.2010.41 PMC4036806

[33] Koutsimani P, Montgomery A, Georganta K. The relationship between burnout, depression, and anxiety: A systematic review and Meta-Analysis. FRONT PSYCHOL. 2019;10:284. 30918490 10.3389/fpsyg.2019.00284 PMC6424886

[34] Elbay RY, Kurtulmus A, Arpacioglu S, Karadere E. Depression, anxiety, stress levels of physicians and associated factors in Covid-19 pandemics. PSYCHIAT RES. 2020;290:113130. 10.1016/j.psychres.2020.113130 PMC7255248 32497969

[35] Appiani FJ, Rodriguez CF, Sarotto L, Yaryour C, Basile ME, Duarte JM. Prevalence of stress, burnout syndrome, anxiety and depression among physicians of a teaching hospital during the COVID-19 pandemic. ARCH ARGENT PEDIATR. 2021;119(5):317–24. 34569739 10.5546/aap.2021.eng.317

[36] Emiral E, Ors B, Canturk N. Assessing depression, anxiety, stress, and occupational decision regret levels among resident physicians working at Ankara university faculty of medicine hospital. TURK J MED SCI. 2024;54(5):970–8. 39473744 10.55730/1300-0144.5875 PMC11518353

[37] Milam LA, Cohen GL, Mueller C, Salles A. The relationship between Self-Efficacy and Well-Being among surgical residents. J SURG EDUC. 2019;76(2):321–8. 30245061 10.1016/j.jsurg.2018.07.028 PMC6380924

[38] Chan MK, Chew QH, Sim K. Burnout and associated factors in psychiatry residents: a systematic review. INT J MED EDUC. 2019;10:149–60. 31381505 10.5116/ijme.5d21.b621 PMC6766386

[39] Elfenbein DM. Confidence crisis among general surgery residents: A systematic review and qualitative discourse analysis. JAMA SURG. 2016;151(12):1166–75. 27603429 10.1001/jamasurg.2016.2792 PMC6941584

[40] Mobarak H, Haddad C, Salameh P, Towair E, El KM, Chatila R. The relationship between self-determination and burnout: mental health outcomes in medical residents. PLoS ONE. 2024;19(12):e308897. 10.1371/journal.pone.0308897 PMC11651559 39689122

